# SRGS: sparse partial least squares-based recursive gene selection for gene regulatory network inference

**DOI:** 10.1186/s12864-022-09020-7

**Published:** 2022-11-30

**Authors:** Jinting Guan, Yang Wang, Yongjie Wang, Yan Zhuang, Guoli Ji

**Affiliations:** 1 Department of Automation, Xiamen University, Xiamen, Fujian China; 2 National Institute for Data Science in Health and Medicine, Xiamen University, Xiamen, Fujian China

**Keywords:** Gene regulatory network, Sparse partial least squares, Recursive gene selection, Single-cell gene expression, Bulk gene expression

## Abstract

**Background:**

The identification of gene regulatory networks (GRNs) facilitates the understanding of the underlying molecular mechanism of various biological processes and complex diseases. With the availability of single-cell RNA sequencing data, it is essential to infer GRNs from single-cell expression. Although some GRN methods originally developed for bulk expression data can be applicable to single-cell data and several single-cell specific GRN algorithms were developed, recent benchmarking studies have emphasized the need of developing more accurate and robust GRN modeling methods that are compatible for single-cell expression data.

**Results:**

We present SRGS, SPLS (sparse partial least squares)-based recursive gene selection, to infer GRNs from bulk or single-cell expression data. SRGS recursively selects and scores the genes which may have regulations on the considered target gene based on SPLS. When dealing with gene expression data with dropouts, we randomly scramble samples, set some values in the expression matrix to zeroes, and generate multiple copies of data through multiple iterations to make SRGS more robust. We test SRGS on different kinds of expression data, including simulated bulk data, simulated single-cell data without and with dropouts, and experimental single-cell data, and also compared with the existing GRN methods, including the ones originally developed for bulk data, the ones developed specifically for single-cell data, and even the ones recommended by recent benchmarking studies.

**Conclusions:**

It has been shown that SRGS is competitive with the existing GRN methods and effective in the gene regulatory network inference from bulk or single-cell gene expression data. SRGS is available at: https://github.com/JGuan-lab/SRGS.

**Supplementary Information:**

The online version contains supplementary material available at 10.1186/s12864-022-09020-7.

## Background

Genes interact and work as gene modules to perform cell functions. Gene regulatory networks (GRNs) describe gene interactions, and GRN identification facilitates the understanding of the underlying molecular mechanism of various biological processes and complex diseases. Besides, GRN inference can facilitate the identification of hub genes or key genes for the considered process or phenotype [1].

Common GRN algorithms developed originally for bulk sample data can be divided into different kinds [2, 3], including those based on correlation (such as WGCNA [4]), information theory (such as ARACNE [5], CLR [6], and MRNET [7]), regression (such as GENIE3 [8]), differential equation [9], Boolean network (such as BoolNet [10]), and Bayesian network (such as bnlearn [11]). With the availability of single-cell RNA sequencing data, it is essential and urgent to infer GRNs from single-cell expression data. Although some GRN methods developed for bulk sample data may be applicable to single-cell data, the distinct characteristic of single-cell expression, i.e., the dropout phenomenon, has driven the appearance of GRN algorithms designed specifically for single-cell data. Many single-cell specific GRN inference methods are based on the same principles as tools developed for bulk data [12], such as PIDC [13] based on information theory, SINCERITIES [14] based on regression, LEAP [15] based on correlation, SCODE [16] based on differential equation, and so on.

Different GRN inference algorithms come with different advantages and disadvantages when dealing with different kinds of expression data. A benchmarking study [17] evaluated GRN algorithms, including five general methods and three single-cell specific methods. They found SCODE performed well on simulated data while not on experimental single-cell data; GENIE3 was the best when being applied to simulated data without dropouts while did not perform well on simulated single-cell data with dropouts. The authors reported that single-cell GRN methods do not necessarily have better performance than general methods and most of the evaluated methods could not predict GRNs accurately from single-cell expression data, even for the ones specifically developed for single-cell data. Another benchmarking study [18] published recently evaluated 12 diverse GRN methods. The authors recommended PIDC, GENIE3 and GRNBoost2 in terms of consistent accuracy on curated models and experimental datasets; GENIE3 and PIDC also had better stability, whereas GRNBoost2 was less sensitive to dropouts; SINCERITIES may become a good choice if being run with accurate imputed pseudotimes. They reported that the overall performance of these approaches was below expectations. The results from benchmarking studies have emphasized the need of developing more accurate and robust GRN modeling methods that are compatible for single-cell expression data.

In this study, to develop a robust GRN algorithm which can be used for both bulk and single-cell expression data, we designed SRGS, SPLS (sparse partial least squares)-based recursive gene selection. Based on SPLS, SRGS can achieve the purpose of regression and feature selection simultaneously. SRGS recursively selects and scores the genes which may have regulations on the considered target gene. To consider the dropout characteristic of single-cell data, we randomly scramble samples, set some expression values to zeroes, and generate multiple copies of data through multiple iterations, making SRGS more robust. We tested SRGS on different kinds of expression data, including simulated bulk data, simulated and experimental single-cell data, and also compared with several existing GRN methods including the ones recommended by benchmarking studies, the ones developed originally for bulk sample data and the ones developed specifically for single-cell gene expression data. It has been shown that SRGS is competitive and robust when dealing with different kinds of datasets.

## Implementation

### SRGS algorithm

Partial least squares (PLS) [19] regression combines features of multiple regression and principal component analysis. Supposed *X* is a *M* × *D* matrix containing *M* observations of *D* genes, *Y* is a *M* × *R* matrix containing *M* observations of *R* response variables, then *X* and *Y* can be decomposed by:

$$X=T{P}^T+E,Y=U{Q}^T+F.$$

where *T* and *U* are *M* × *K* score matrices (component scores or hidden variables) of *X* and *Y* respectively, *K* is the number of hidden factors*, P* and *Q* are *D* × *K* and *R* × *K* orthogonal loading matrices, and *E* and *F* are the residual matrices. The decompositions of *X* and *Y* are made so as to maximize the covariance between *T* and *U*. Then based on *T*, *P*, *U*, and *Q*, we can first fit *U* and *T*, then the linear relationship between *X* and *Y* can be obtained. Compared with PLS, sparse partial least squares (SPLS) [20] can not only perform regression but also achieve the purpose of feature selection, for which a regularization parameter *λ* is used to achieve sparsity.

As a larger regularization parameter *λ* of SPLS results in less returned genes, SPLS will return more and more important genes when *λ* is increasing. Therefore, we designed a recursive procedure to select important regulatory genes and measure their influences on a target gene for constructing GRNs, i.e., SPLS-based recursive gene selection (SRGS). For each considered target gene *i*, SRGS recursively selects and scores the genes which may have regulations on gene *i*. The expression of gene *i* is denoted as *Y*, and the expression of other genes (whose number is denoted as *d*) is denoted as *X*. In SRGS, the regularization parameter *λ* of SPLS is increased recursively. Given the current *λ*, *Y*, and *X*, SPLS selects the genes which may have regulation on gene *i*. If the number of the selected genes is greater than zero and less than *d*, the scores of these selected genes are added by one. At the end of recursion, the cumulative scores of the *d* genes are normalized using min-max normalization and then put into the *i*-th row of a score matrix. The above process is repeated for each gene for obtaining the score matrix to describe gene relationships. Noted that the score matrix may be not symmetry and SRGS can be regarded as a method that can describe directional regulatory relationships.

Compared with bulk RNA-seq data, a sample of single-cell gene expression data represents a cell and experimental single-cell gene expression data contains a large number of zeroes including true and false zeroes, i.e., dropout phenomenon, which challenges the identification of gene regulatory networks. In order to make SRGS robust and applicable to single-cell gene expression data, we perform random permutations for samples, set some values in the expression matrix *X* to zeroes, and generate multiple copies of data through multiple iterations. We use a parameter 𝛿_*jk*_, whose value is either 0 or 1, to indicate whether the expression level of a gene *k* in the *j*-th sample is set to zero or not. Whether the gene expression is set to zero or not is subject to the Bernoulli distribution of parameter *p*, *p* ∈ [0,1], that is, P(𝛿_*jk*_ = 1) = *p*.

### Simulated datasets and ground-truths

GeneNetWeaver (GNW) [21] is commonly applied to generate simulated gene expression data and evaluate GRN modeling methods. To obtain a reference network, GNW extracts the topology of a subnetwork from the transcriptional regulatory networks that were derived from experimental data, and then expression datasets are generated by simulations based on stochastic differential equations. GNW has been used in several Dialogue for Reverse Engineering Assessments and Methods (DREAM) network inference challenges.

Firstly, we used DREAM3 network challenge data as simulated bulk expression data and corresponding gold-standard networks. DREAM3 datasets consist of three differently sized networks including 10, 50, and 100 genes, and for each gene size there are five gold-standard networks including Ecoli1, Ecoli2, Yeast1, Yeast2, and Yeast3. For each true network, three different types of data are provided, including heterozygous knockdown, null-mutant knockout, and time series data. In this study, knockout data is used.

Secondly, to mimic true single-cell gene expression data, we applied GNW to simulate 1000 time-series experiments for all networks with gene sizes of 50 and 100, using the default settings and the default time points: times 0 to 1000, in steps of 50. Then based on these 1000 time-series data, we generated datasets with different numbers of samples including 10, 50, 100, 500, 1000 and 5000. We sampled a single time point from each time series, representing a single cell. For the dataset with 5000 cells, we sampled 250 cells from time 0 onwards in time steps of 50; for the dataset with 1000 cells, we sampled 50 cells from time 0 onwards in time steps of 50; for the dataset with 500 cells, we sampled 50 cells from time 0 onwards in time steps of 100; for the dataset with 100 cells, we sampled 10 cells from time 0 onwards in time steps of 100; for the dataset with 50 cells, we sampled 25 cells from time 0 onwards in time steps of 500; for the dataset with 10 cells, we sampled 5 cells from time 0 onwards in time steps of 500.

Lastly, considering the dropout events of experimental single-cell expression data, to test the robustness of GRN methods to single-cell data with dropouts, we further simulated dropout datasets based on the four simulated single-cell expression data from GNW, including the ones containing 50 genes and 50 samples, 100 genes and 50 samples, 50 genes and 100 samples, and 100 genes and 100 samples. We induced dropout events to these mimic single-cell expression data at three rates, such that expression values in the lowest 20% (low rate), 50% (medium rate), or 70% (high rate) for each gene would be replaced as zeroes according to a Bernoulli distribution probability of 0.5.

### Experimental single-cell datasets and ground-truths

We used four experimental single-cell RNA-seq datasets to test GRN inference algorithms:

1. Mouse hematopoietic stem cell dataset (mHSC) [22], which contains three lineages, erythroid, granulocyte-monocyte and lymphoid. GRNs were inferred for each lineage independently, which is denoted as mHSC-E, mHSC-GM and mHSC-L.
2. Mouse dendritic cell dataset (mDC) [23], which corresponds to bone-marrow derived dendritic cells under various conditions. The lipopolysaccharide stimulated wildtype cells measured at 1, 2, 4 and 6 h were used in this study.
3. Human mature hepatocyte dataset (hHEP) [24], which was derived from induced pluripotent stem cells in two-dimensional culture differentiating to hepatocyte-like cells. It contains measurements from multiple time points.
4. Human embryonic stem cell dataset (hESC) [25], which was derived along the differentiation protocol to produce definitive endoderm cells from human embryonic stem cells.

The processed datasets and the computed pseudotimes, gene variances and associated *P*-values were obtained from a benchmarking study [18]. Firstly, we used the Bonferroni method to correct for multiple tests and calculated the corrected *P*-values. Then we selected top 500 and 1000 variable genes for GRN inference, which are genes whose variances rank top 500 and corrected *P*-values are less than 0.01 and genes whose variances rank top 1000 and corrected *P*-values are less than 0.01.

After applying a GRN inference algorithm, we only evaluated the interactions outgoing from a transcription factor (TF). To evaluate GRN methods, three types of ground-truths were used as references, including cell-type-specific ChIP-seq data, nonspecific ChIP-seq data and functional interaction network. Cell-type-specific ChIP-seq data was collected from the same or similar cell type as the experimental scRNA-seq dataset, searched from ENCODE, ChIP-Atlas and ESCAPE databases. Nonspecific ChIP-seq data consists of (1) ChIP-seq and transcriptional regulatory information with curated/highly confident and likely confident evidence in the database of DoRothEA [26] (2); TF-TF and TF-gene interactions for human and mouse in RegNetwork [27] (3); TF-target interactions for human and mouse in TRRUST [28]. Human and mouse STRING [29] networks were used as functional interaction networks. The list of TFs and ground-truths were downloaded from the benchmarking study [18].

### Comparisons with existing GRN algorithms

We compared SRGS with ten different GRN algorithms: (1) regression-based methods including GENIE3 [8], and SINCERITIES [14] (2); information theoretic methods including ARACNE [5], CLR [6], MRNET [7], and PIDC [13] (3); correlation-based methods including PPCOR [30] and LEAP [15] (4); Boolean network-based method, BoolNet [10]; and (5) Bayesian network-based method, bnlearn [11]. Among them, SINCERITIES, PIDC, and LEAP were specifically developed for single-cell expression data. After obtaining the correlations between genes from each GRN method, the area under receiver operating characteristic curve (denoted as AUROC) and the area under precision and recall curve (denoted as AUPR) are calculated as indicators to assess the performance. For this, we converted the inferred correlations between genes and the ground-truth respectively to edge lists including all gene pairs, ignoring self-loops. Then the two edge lists are compared and the cutoff of correlation value is varied to calculate each point of ROC or PR curve.

## Results

### Overall analyses

**Fig. 1:**
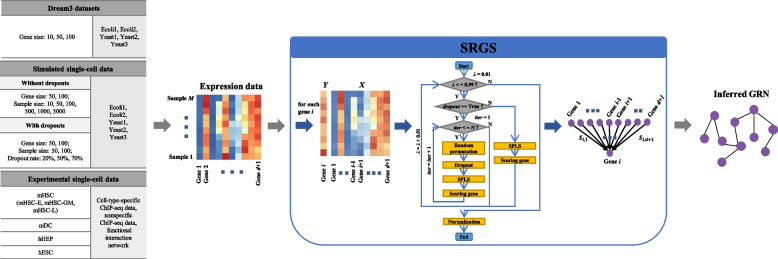
The overall analytical workflow, which lists the used datasets and corresponding referenced ground-truths, and describes the procedure of SRGS

We propose SRGS, SPLS (sparse partial least squares)-based recursive gene selection, for GRN inference. The overall analytical workflow is shown in Fig. 1. For each considered target gene *i*, SRGS recursively selects and scores the genes which may have regulations on this considered gene based on SPLS by increasing the regularization parameter *λ* of SPLS recursively. The parameter of *K* of SPLS can be specified by users. When dealing with gene expression data with dropouts, we perform multiple iterations (whose number is denoted by *iter*) to generate multiple copies of data, for each of which samples are randomly permutated, some values in the expression matrix *X* are set to zeroes, and then SPLS is used to score genes. The probability that the gene expression is kept is subject to the Bernoulli distribution of the parameter *p*, *p* ∈ [0,1]. At the end of recursion, the cumulative scores are normalized. After the score matrix is obtained, the area under receiver operating characteristic curve (denoted as AUROC) and the area under precision and recall curve (denoted as AUPR) are used as indicators to assess the performance of SRGS. To test SRGS, we used DREAM3 dataset to mimic bulk gene expression data, and used simulated GNW time-series data without and with dropouts to mimic single-cell gene expression data. In addition, we also performed SRGS on experimental single-cell datasets. We compared SRGS with ten existing GRN algorithms including GENIE3, SINCERITIES, ARACNE, CLR, MRNET, PIDC, PPCOR, LEAP, BoolNet, and bnlearn. These GRN methods contain the ones recommended by benchmarking studies, such as GENIE3 and PIDC, the one developed originally for bulk data, and the ones developed specifically for single-cell data.

### SRGS on DREAM3 datasets

**Fig. 2:**
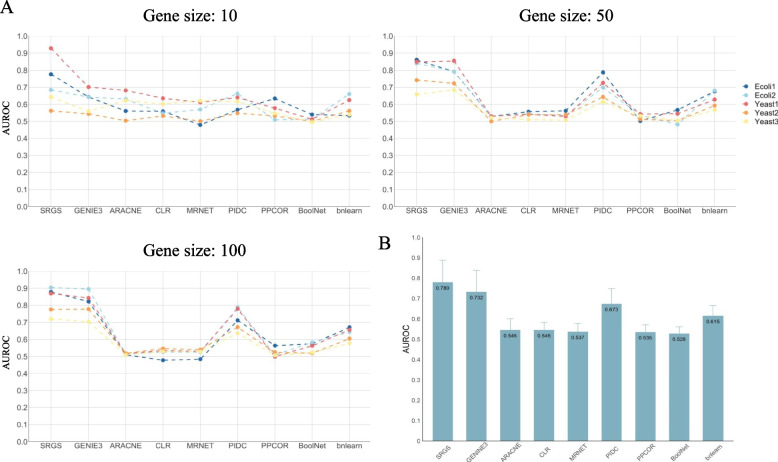
**A** The performance index, AUROC, of SRGS and each compared GRN method tested on DREAM3 datasets. The DREAM3 datasets contain three different gene sizes (10, 50 and 100) and five gold-standard networks (Ecoli1, Ecoli2, Yeast1, Yeast2, and Yeast3). **B** The mean and standard deviation of AUROC of each GRN method across all gene sizes, sample sizes and networks shown in (**A**). The benchmarking methods are GENIE3, ARACNE, CLR, MRNET, PIDC, PPCOR, BoolNet, and bnlearn

The DREAM3 knockout datasets of all gene sizes (10, 50 and 100) were used, and all kinds of gold-standard networks (Ecoli1, Ecoli2, Yeast1, Yeast2, and Yeast3) were used as references. In this case, we only tested the methods which do not need pseudotime information, i.e., SRGS, GENIE3, ARACNE, CLR, MRNET, PIDC, PPCOR, BoolNet, and bnlearn. For SRGS, when it was tested on bulk expression datasets, we did not perform random permutations and generate multiple copies of data. We just recursively increased the regularization parameter *λ* of SPLS and scored the genes which may have regulations on each considered target gene, which means the parameter of *dropout* was set to FALSE in the workflow of SRGS shown in Fig. 1. To consider the effect of parameter *K* of SPLS, and the range and step size of *λ* on the performance of SRGS, we took the Ecoli1 dataset with gene size of 50 as an example and used AUROC as a performance index. The relation between AUROC and *K* is shown in Supplementary Fig. 1. A, which indicates the optimal value of *K* is one; therefore, we used one as the default value of *K*. We compared three ranges of *λ* using a fixed step size of 0.01, including from 0.01 to 0.99 (AUROC: 0.861), from 0.01 to 0.5 (AUROC: 0.836), and from 0.5 to 0.99 (AUROC: 0.854), and found the performance using the range from 0.01 to 0.99 was better than other two ranges. Then we set *λ* as from 0.01 to 0.99 and compared two step sizes, 0.01 (AUROC: 0.861) and 0.1 (AUROC: 0.854), and found that the performance of SRGS is better when using a small step size. Therefore, we set the default range of *λ* as from 0.01 to 0.99 and the step size as 0.01. Figure 2. A and Supplementary Table 1 show the AUROC and AUPR values of all methods tested on different datasets. We then calculated the average and standard deviation of AUROC (Fig. 2. B) and AUPR (Supplementary Fig. 2. A) values of each method and also performed two-sided paired *t*-tests to compare SRGS with each of other methods (Supplementary Table 1). It can be noted that SRGS shows the best performance overall, and then GENIE3, PIDC and bnlearn. SRGS is outstanding across different datasets (*p*-value of *t*-test < 0.1) and is competitive with the existing GRN methods no matter based on AUROC or AUPR.

### SRGS on simulated single-cell datasets

**Fig. 3:**
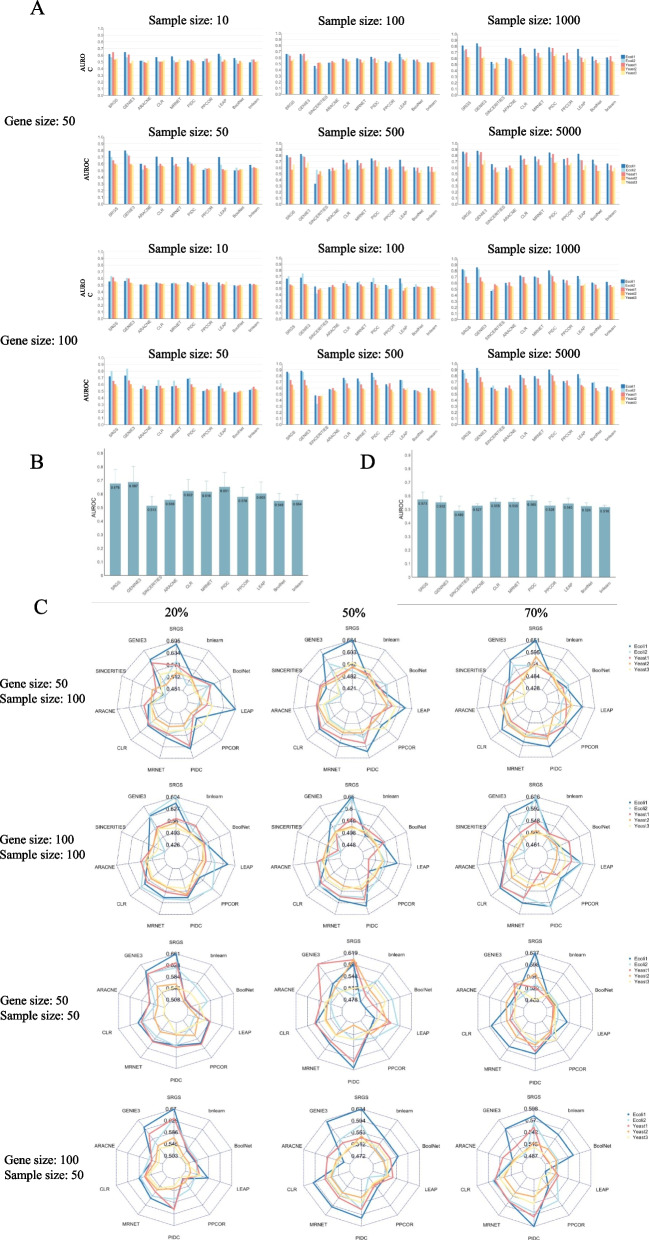
**A** The AUROC values of SRGS and each compared GRN method tested on simulated single-cell datasets without dropouts. The datasets contain two gene sizes (50 and 100) and six sample sizes (10, 50, 100, 5,001,000 and 5000). For each dataset, five gold-standard networks (Ecoli1, Ecoli2, Yeast1, Yeast2, and Yeast3) were used as references. **B** The mean and standard deviation of AUROC of each GRN method across all gene sizes, sample sizes and networks shown in (**A**). **C** The AUROCs of SRGS and each compared GRN method tested on simulated single-cell datasets with dropouts. The datasets contain two gene sizes (50 and 100), two sample sizes (50 and 100), and three dropout rates (20, 50, and 70%). For each dataset, five gold-standard networks (Ecoli1, Ecoli2, Yeast1, Yeast2, and Yeast3) were used as references. **D** The mean and standard deviation of AUROC of each GRN method across all gene sizes, sample sizes, networks, and dropout rates shown in (**C**). The benchmarking methods are GENIE3, SINCERITIES, ARACNE, CLR, MRNET, PIDC, PPCOR, LEAP, BoolNet, and bnlearn. For SINCERITIES, the datasets with sample sizes of 10 and 50 could not be tested

To test SRGS on simulated single-cell gene expression data, we first used GNW to generate 1000 time-series experiments for all networks with gene sizes of 50 and 100. Then based on these 1000 time-series data, we used a single time point from each time series to represent a single cell and generated datasets with different numbers of samples including 10, 50, 100, 500, 1000 and 5000. These simulated datasets without dropouts were not sparse; hence, we continued to set the parameter of *dropout* to FALSE in the workflow of SRGS. To consider the effect of parameter *K*, and the range and step size of *λ* on the performance of SRGS, we took the Ecoli1 dataset with gene size of 50 and sample size of 500 as an example. The relation between AUROC and *K* is shown in Supplementary Fig. 1. B, which also shows the optimal value of *K* is one. We compared three ranges of *λ*, from 0.01 to 0.99 (AUROC: 0.805), from 0.01 to 0.5 (AUROC: 0.791), and from 0.5 to 0.99 (AUROC: 0.748), and compared two step sizes, 0.01 (AUROC: 0.805) and 0.1 (AUROC: 0.794). It was also found that the performance of SRGS is better when using a bigger range and a smaller step size, so we kept setting the range of *λ* as from 0.01 to 0.99 and the step size as 0.01. On these simulated datasets, we compared SRGS with other ten GRN methods using performance indexes AUROC (Fig. 3. A) and AUPR (Supplementary Table 1). Overall, GENIE3 is the best, and then SRGS and PIDC. SRGS can be better than PIDC in many datasets. To show the overall performance of the evaluated GRN methods, we calculated the means and standard deviations of AUROCs (Fig. 3. B) and AUPRs (Supplementary Fig. 2. B) across all datasets and also performed two-sided paired *t*-tests to compare SRGS with each of other methods (Supplementary Table 1). It can be seen that GENIE3, SRGS and PIDC are the top three methods across all datasets. Based on AUROC, SRGS is better than PIDC, while based on AUPR, PIDC is slightly better than SRGS.

The simulated single-cell gene expression data without dropouts can be seen as true single-cell data, while experimental single-cell gene expression data are characterized by dropout phenomenon, which hinders the identification of gene regulatory networks. Therefore, to consider the dropout feature of single-cell expression data, we further induced dropout events to four sizes of simulated single-cell expression datasets from GNW, including the ones containing 50 genes and 50 samples, 100 genes and 50 samples, 50 genes and 100 samples, and 100 genes and 100 samples. The dropout events were induced at three rates, 20% (low rate), 50% (medium rate), and 70% (high rate). When testing on single-cell datasets with dropouts, we set the parameter of *dropout* to TRUE, meaning that we performed random permutation for samples, set some values in the expression matrix to zeroes, and generated multiple data copies through multiple iterations. The probability that the gene expression is kept is subject to the Bernoulli distribution of the parameter *p*, *p* ∈ [0,1], and *p* = 1 actually means that we do not generate multiple datasets. Firstly, to show the effectiveness of *p*, taking the Ecoli1 datasets with sample size of 100 and gene sizes of 50 and 100 as examples and using AUROC as a performance index, we varied the value of *p* from 0.1 to 1 with a step size of 0.1, repeated the procedure for ten times, and calculated the mean of AUROCs among the ten times (Supplementary Fig. 3. A). We found that compared with *p* = 1, setting *p* as an appropriate value can make SRGS achieve better performance, which indicates the dropout processing and generation of multiple copies can make SRGS more robust. Besides, for the Ecoli1 dataset with 20, 50 and 70% dropout rates, we could determine the optimal *p* as 0.9, 0.6 and 0.6 respectively, for gene sizes 50 and 100. Then, we used the Ecoli1 dataset with sample size of 100, gene size of 50 and dropout rate of 50% as an example to check the effect of the number of iterations (*iter*) on the performance of SRGS. We used its optimal *p* and compared different values of *iter*, 1, 10, 50 and 100 (Supplementary Fig. 3. B). It was found that ten times of iterations are enough, which can also reduce the running time. Therefore, we used the selected *p* (0.9, 0.6, and 0.6 for 20%, 50%, and 70% dropout rates) and set *iter* as ten for all datasets to ran SRGS. Fig. 3. C and Supplementary Table 1 show the AUROC and AUPR values of all methods tested on different datasets. To show the overall performance of all methods, we calculated the mean and standard deviation of each method across all datasets (Fig. 3. D and Supplementary Fig. 2. C). The AUPRs of all methods are very small and many of them are not discriminative. Based on AUPR, MRNET is the best one and also the only one method significantly better than SRGS (difference = 0.004, *p*-value of *t*-test < 0.1). According to AUROC, we noted that on the simulated single-cell datasets with dropouts, PIDC shows its advantage compared with GENIE3, while SRGS can still demonstrate a competitive performance. SRGS, PIDC, CLR and MRNET are the top methods (Fig. 3. D), while GENIE3, the best one on simulated single-cell datasets without dropouts (Fig. 3. B), did not perform well in this case. As to SINCERITIES, we noticed that when sample size is larger or time step is smaller (such as the sample size of 5000 in Fig. 3. A), the performance of SINCERITIES would be better, and it may be also affected by the existence of dropouts (Fig. 3. C). The dropout phenomenon of single-cell gene expression data challenges the identification of gene regulatory networks, the performances of most GRN methods are below expectations, which is also emphasized in the recent benchmarking studies [17, 18].

### SRGS on experimental single-cell datasets

Four experimental single-cell transcriptomic data including two mouse and two human datasets (mDC, mHSC, hESC, and hHEP) were analyzed. For the mHSC dataset, GRNs were inferred for each lineage independently, denoted as mHSC-E, mHSC-GM and mHSC-L. The numbers of samples and genes can be seen in Supplementary Table 2. We selected the top 500 and 1000 variable genes for GRN inference. The variances and adjusted *P*-values of top variable genes are listed in Supplementary Table 3. We used three kinds of ground-truths including cell-type-specific ChIP-seq data, nonspecific ChIP-seq data and STRING functional interaction network. After applying a GRN inference algorithm, we only evaluated the interactions outgoing from a transcription factor (TF). The corresponding numbers of genes and TFs for each single-cell dataset and each ground-truth, and the numbers of overlapping genes and TFs between the top genes and referenced ground-truths can be seen in Supplementary Table 2.

**Fig. 4:**
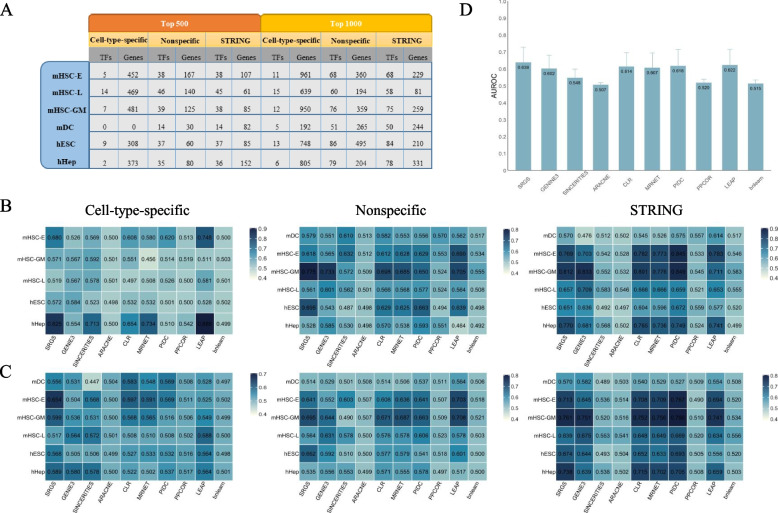
The AUROCs of SRGS and each compared GRN method tested on experimental single-cell gene expression datasets, including mHSC-E, mHSC-GM, mHSC-L, mDC, hHEP, and hESC. The referenced ground-truths include cell-type-specific ChIP-seq data, nonspecific ChIP-seq data, and STRING functional interaction network. The top 500 or 1000 variable genes were used for GRN inference. **A** The number of overlapping TFs between the top genes in each single-cell dataset and the regulators in each referenced ground-truth, and the number of overlapping genes between the top genes in each single-cell dataset and the targets in each referenced ground-truth. The AUROCs of all methods tested on all datasets and ground-truths, when (**B**) top 500 genes and (**C**) top 1000 genes were used for GRN inference. **D** The mean and standard deviation of AUROC of each GRN method across all single-cell datasets, referenced ground-truths, and different numbers of top genes. The evaluated methods are SRGS, GENIE3, SINCERITIES, ARACNE, CLR, MRNET, PIDC, PPCOR, LEAP, and bnlearn

Figure 4. A lists the number of overlapping TFs between the top genes in each single-cell dataset and the regulators in each referenced ground-truth, and the number of overlapping genes between the top genes in each single-cell dataset and the targets in each referenced ground-truth. We first selected the optimal value of Bernoulli distribution parameter *p* for each dataset using AUROC as a performance index (Supplementary Fig. 4), and then used the selected *p* to run SRGS. Considering the problem of running speed, we had not tested BoolNet. Figure 4. B and C show the AUROCs of all methods tested on all datasets and ground-truths when top 500 and 1000 variable genes were used for GRN inference. Supplementary Table 1 lists AUPR values of all methods. We also calculated the mean and standard deviation across all data tests to show the overall performance of each method (Fig. 4. D and Supplementary Fig. 2. D). Each method has a small AUPR and even a larger standard deviation than the mean. Based on AUROC, SRGS, LEAP and PIDC are the top three methods. When being tested on experimental single-cell datasets, no single method can stand out, while several methods show better performances on some datasets. For the hHep dataset, when top 500 genes and cell-type-specific ChIP-seq data were used, LEAP and SRGS can achieve AUROC of 0.8; for mHSC-GM, when top 500 genes and nonspecific ChIP-seq data were used, the AUROCs of SRGS and GENIE3 can achieve 0.78 and 0.73; also for mHSC-GM, when top 1000 genes and nonspecific ChIP-seq data were used, the AUROCs of SRGS and LEAP are around 0.7. When STRING network is used as a reference, more methods can show better performances compared with the cases when cell-type-specific and nonspecific ChIP-seq data are used, especially PIDC. In this case, PIDC shows the best AUROC on mHSC-E and mHSC-GM, and SRGS is the best on hHep dataset no matter using top 500 or 1000 genes. Compared with the results on the simulated single-cell data with dropouts, the performances of GRN methods on experimental single-cell data are better. This is likely because that the percentage of dropouts of the tested experimental datasets is not as high as the simulated single-cell data with dropouts.

With regard to the selection of the Bernoulli distribution parameter *p*, we noticed that for almost all datasets, when a specific referenced ground-truth and a gene expression dataset are given, the optimal value of *p* is the same no matter using top 500 or 1000 genes (Supplementary Fig. 4). Then we checked if we could use less genes and even less samples to determine the optimal value of *p*. Using AUROC as a performance index, we took the mHSC-E as an example to examine the optimal *p* when using top 1000 genes, top 500 genes, top 300 genes, and using top 300 gene and also randomly selected 300 samples (Supplementary Fig. 5). The optimal *p* could be the same. This may provide users a way to choose an appropriate value of *p*. Besides, if users would like to choose the exact optimal value of *p*, or test on large gene expression datasets, we provide parallel computing, accelerating the running of SRGS.

## Discussion

With the availability of more and more single-cell RNA sequencing data, it is of significance to infer GRNs from single-cell gene expression. Although some GRN methods developed for bulk sample data could be applicable to single-cell data, single-cell expression has its distinct characteristics and the development of GRN algorithms designed for single-cell data is needed. Although several single-cell specific GRN methods were proposed, while the recent benchmarking studies have shown single-cell GRN methods do not necessarily have better performance than general methods. It is still needed and challengeable to develop more accurate and robust GRN modeling methods that can be used for single-cell gene expression data.

In this study, we developed a GRN algorithm, SRGS, based on SPLS and recursive gene selection. We tested SRGS on different kinds of expression datasets, including simulated bulk data, simulated single-cell data without and with dropouts, and experimental single-cell data. We compared SRGS with exiting GRN methods, including the ones developed originally for bulk sample data, the ones developed specifically for single-cell gene expression data, and the ones recommended by recent benchmarking studies. When testing on DREAM3 data and simulated single-cell data without dropouts, we recursively increased the regularization parameter of SPLS and scored the genes which may have regulations on each considered target gene, as these datasets were not sparse. On DREAM3 datasets, SRGS is the best, and then GENIE3 and PIDC; on simulated single-cell data without dropout, GENIE3 is the best and then SRGS and PIDC. When testing on simulated single-cell data with dropouts and experimental datasets, we performed multiple iterations to generate multiple copies of data, for each of which samples were randomly scrambled, and some values in the expression matrix were dropped out. On the simulated single-cell datasets with dropouts, SRGS, PIDC, CLR and MRNET are the top methods. PIDC shows its advantage compared with GENIE3 which is not robust enough from data without dropouts to with dropouts, while SRGS can still demonstrate a competitive performance. On experimental single-cell datasets, SRGS, LEAP and PIDC rank top three. These results show that SRGS is competitive with the existing GRN modeling methods, even the ones recommended by benchmarking studies (i.e., GENIE3 and PIDC). Moreover, SRGS provides parallel computing when being faced with a large gene expression dataset. SRGS is robust and can be used for bulk and single-cell expression data.

## Conclusion

We developed a sparse partial least squares-based recursive gene selection method, SRGS, for gene regulatory network inference. SRGS was tested on simulated bulk data, simulated single-cell data with and without dropouts, and experimental single-cell data, and also compared with existing GRN methods. It has been shown that SRGS is competitive and effective, and can be used for GRN inference from bulk or single-cell gene expression data.

### Availability and requirements

• Project name: SRGS

• Project home page: https://github.com/JGuan-lab/SRGS

• Operating system(s): Platform independent

• Programming language: R

• Other requirements: R 3.6.3 or higher

• License: GNU GPL

• Any restrictions to use by non-academics: None

## Supplementary Information

**Additional file 1: Supplementary Table 1.** The performance indexes, AUROC and AUPR, of SRGS and each compared GRN method tested on DREAM3 datasets, simulated single-cell datasets without dropouts, simulated single-cell datasets with dropouts, and experimental single-cell gene expression datasets. The difference of means of AUROC and AUPR between SRGS and each of other methods and the *p*-value of two-sided paired *t*-test are also listed. For SINCERITIES, the datasets with sample sizes of 10 and 50 could not be tested.

**Additional file 2: Supplementary Table 2.** The numbers of genes and TFs for each single-cell dataset and each ground-truth, and the numbers of overlapping genes and TFs between the referenced ground-truths and the top genes.

**Additional file 3: Supplementary Table 3.** The variances and adjusted *P*-values of top variable genes which were used for GRN inference.

**Additional file 4: Supplementary Fig. 1.** The effect of parameter *K* of SPLS on the performance of SRGS. (A) The DREAM3 Ecoli1 dataset with gene size of 50 was used as an example to test SRGS. (B) The Ecoli1 dataset with gene size of 50 and sample size of 500 was used as a representative of simulated single-cell datasets without dropouts to test SRGS.

**Additional file 5: Supplementary Fig. 2.** The mean and standard deviation of AUPR of each GRN method across all data tests of (A) DREAM3 datasets, (B) simulated single-cell datasets without dropouts, (C) simulated single-cell datasets with dropouts, and (D) experimental single-cell gene expression datasets.

**Additional file 6: Supplementary Fig. 3.** (A) The effect of the Bernoulli distribution parameter *p* on the performance of SRGS was examined. The Ecoli1 datasets with sample size of 100, two different gene sizes (50 and 100) and three different dropout rates (20, 50, and 70%) were used as representatives of simulated single-cell datasets with dropouts. The value of *p* was varied from 0.1 to 1 with a step size of 0.1, and the procedure was repeated for ten times. The mean of AUROCs among the ten times was shown. (B) The Ecoli1 dataset with sample size of 100, gene size of 50 and dropout rate of 50% was used as an example to check the effect of the number of iterations on the performance of SRGS.

**Additional file 7: Supplementary Fig. 4.** The selection of Bernoulli distribution parameter *p* for each experimental single-cell gene expression dataset (including mHSC-E, mHSC-GM, mHSC-L, mDC, hESC and hHEP) and each referenced ground-truth (including cell-type-specific ChIP-seq data, nonspecific ChIP-seq data, and STRING network) when top 500 and 1000 genes were used for GRN inference.

**Additional file 8: Supplementary Fig. 5.** The selection of Bernoulli distribution parameter *p* when top 300 genes and also randomly selected 300 samples, top 300 genes, top 500 genes, and top 1000 genes were used. mHSC-E was used as a representative of experimental single-cell gene expression datasets to test.

## Data Availability

SRGS is available at GitHub: https://github.com/JGuan-lab/SRGS. The analysed DREAM3 datasets, simulated datasets, and the processed experimental single-cell datasets can also be accessed at GitHub: https://github.com/JGuan-lab/SRGS. The experimental datasets and corresponding ground-truths were originally downloaded from a benchmarking study of Pratapa et al. at Zenodo: 10.5281/zenodo.3701939.

## Abbreviations

GRN: Gene regulatory network
SPLS: Sparse partial least squares
GNW: GeneNetWeaver
DREAM: Dialogue for Reverse Engineering Assessments and Methods
mHSC: Mouse hematopoietic stem cell
mDC: Mouse dendritic cell
hHEP: Human mature hepatocyte
hESC: Human embryonic stem cell
TF: Transcription factor
AUROC: Area under receiver operating characteristic curve
AUPR: Area under precision and recall curve
