# Supplementary figures and images for: SRGS: sparse partial least squares-based recursive gene selection for gene regulatory network inference
Source: BMC Genomics. 2022 Nov 30;23:782. doi: 10.1186/s12864-022-09020-7 (PMC9710113; doi:10.1186/s12864-022-09020-7)

A

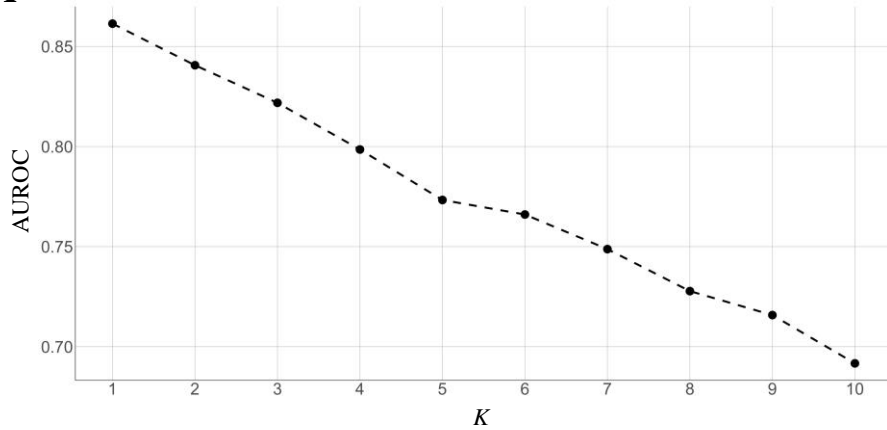

B

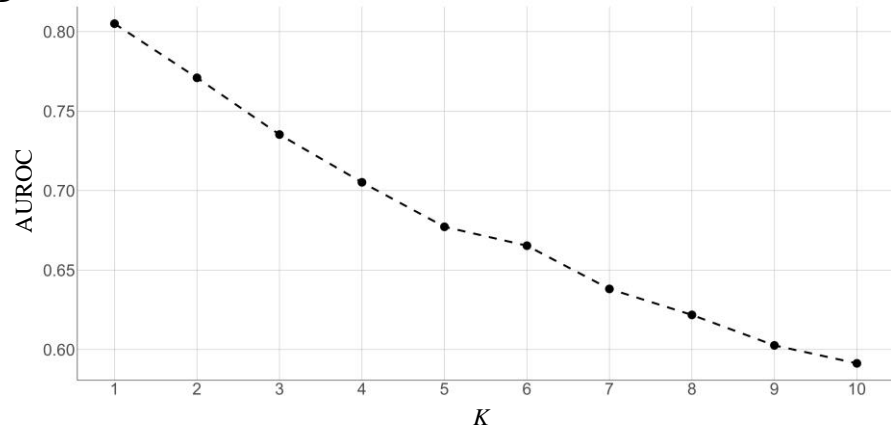

Supplement: Supplementary file 4 — Additional file 4: Supplementary Fig. 1. The effect of parameter K of SPLS on the performance of SRGS. (A) The DREAM3 Ecoli1 dataset with gene size of 50 was used as an example to test SRGS. (B) The Ecoli1 dataset with gene size of 50 and sample size of 500 was used as a representative of simulated single-cell datasets without dropouts to test SRGS. [file 12864_2022_9020_MOESM4_ESM.pdf]

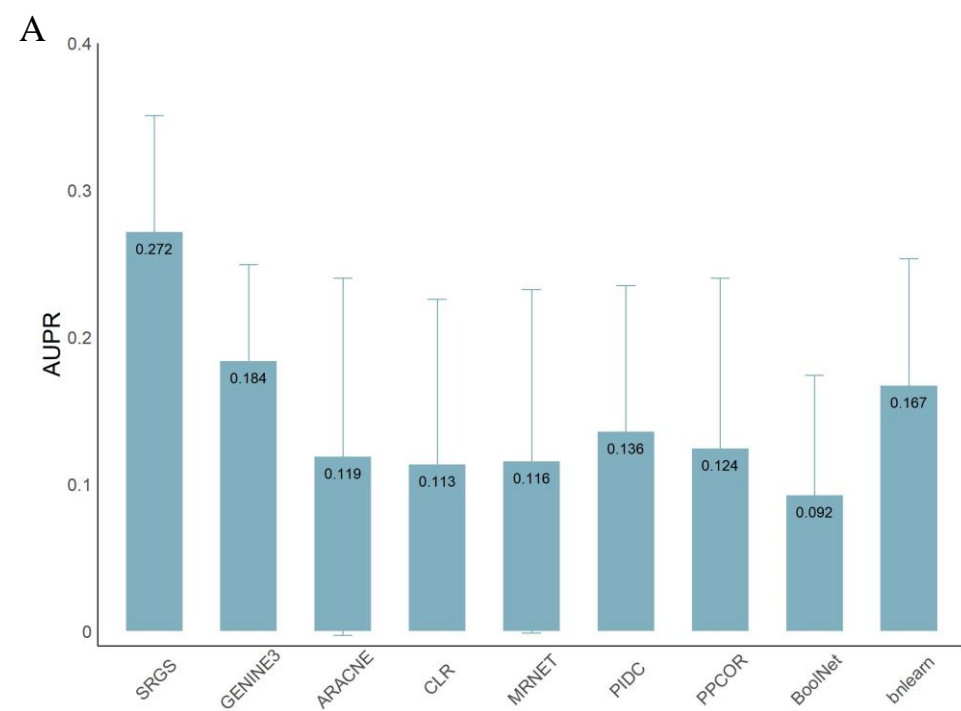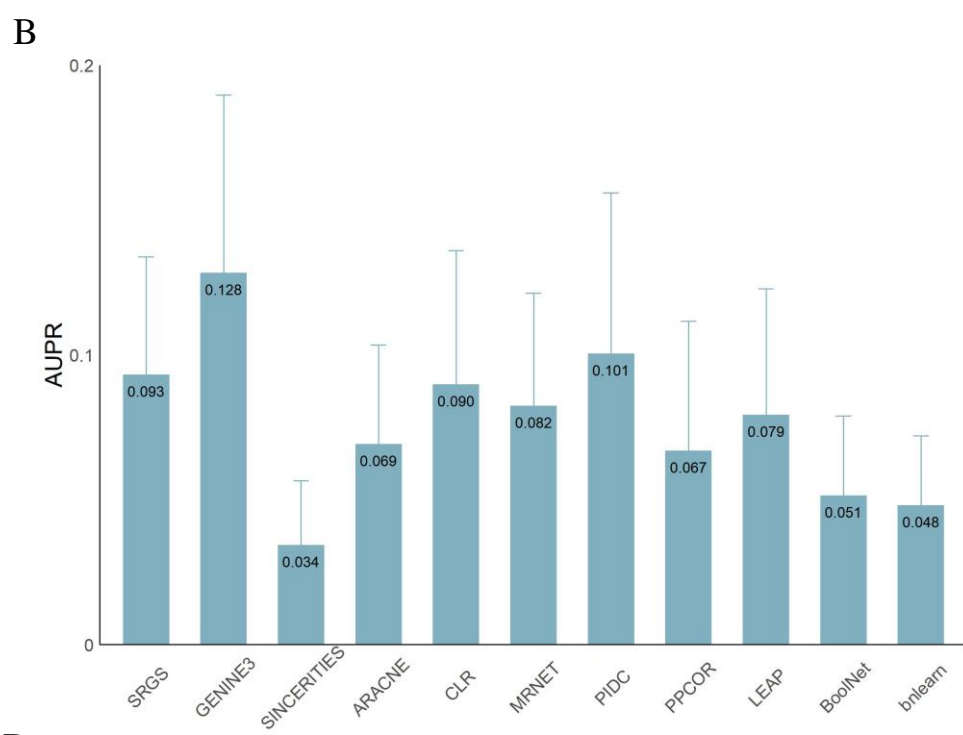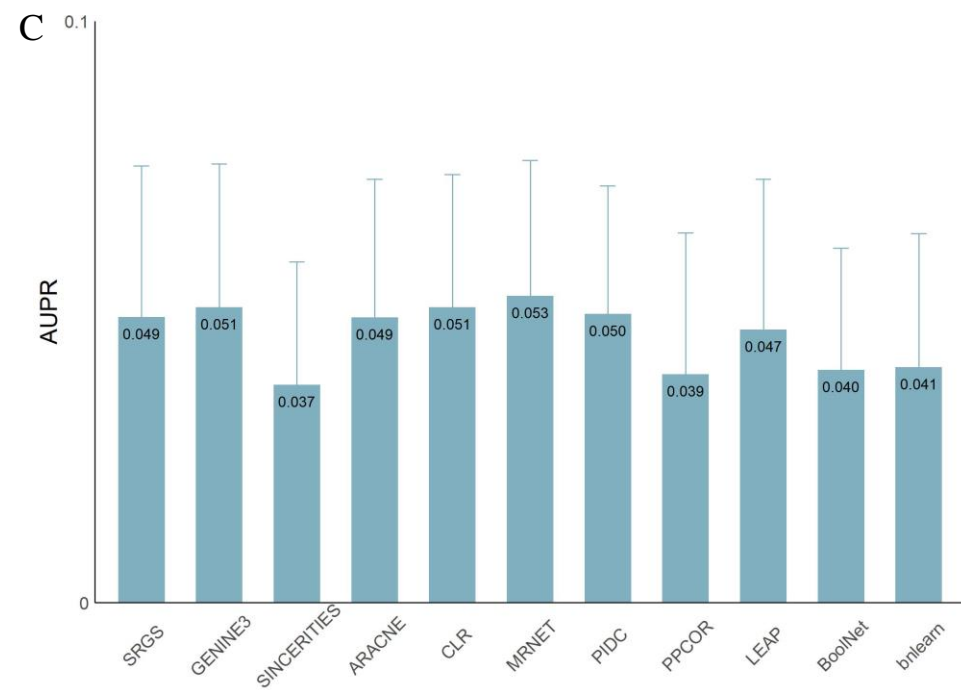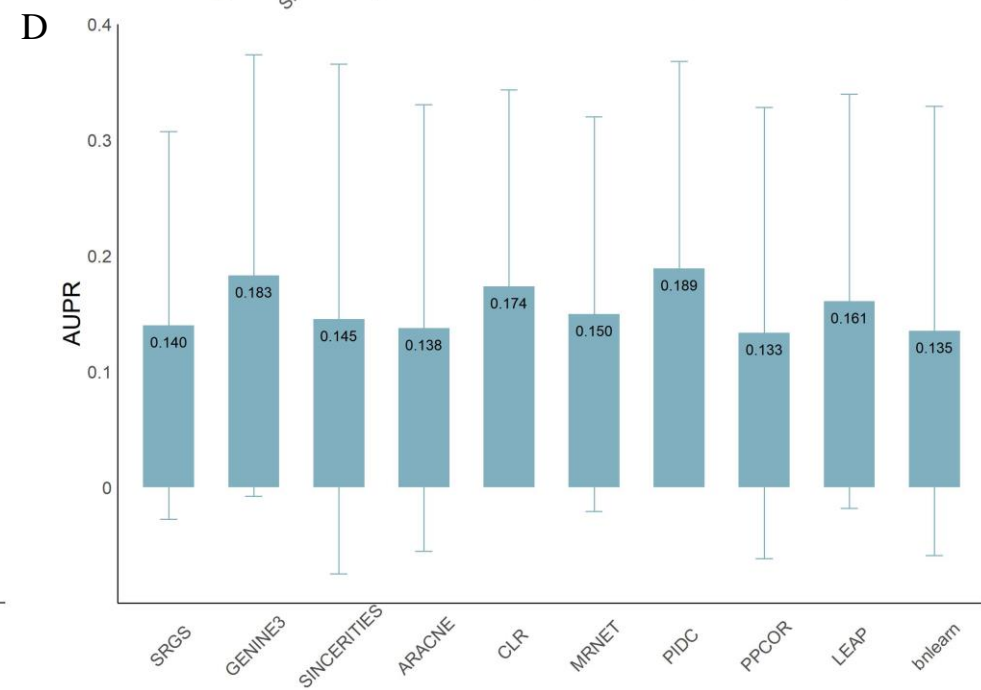

Supplement: Supplementary file 5 — Additional file 5: Supplementary Fig. 2. The mean and standard deviation of AUPR of each GRN method across all data tests of (A) DREAM3 datasets, (B) simulated single-cell datasets without dropouts, (C) simulated single-cell datasets with dropouts, and (D) experimental single-cell gene expression datasets. [file 12864_2022_9020_MOESM5_ESM.pdf]

A

gene size: 50

gene size: 100

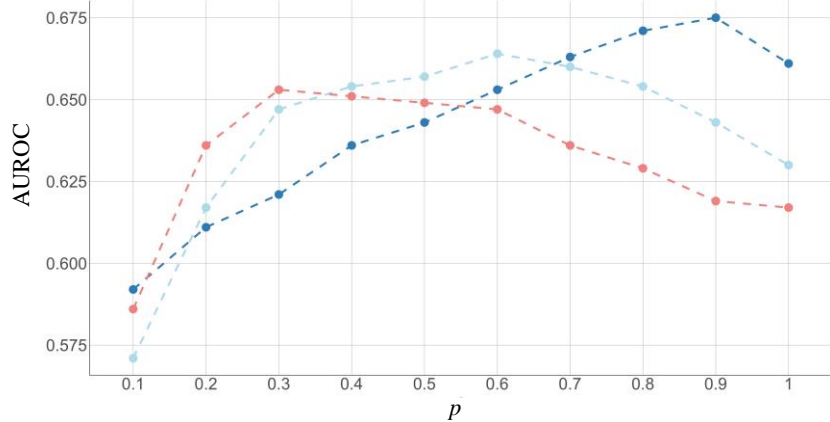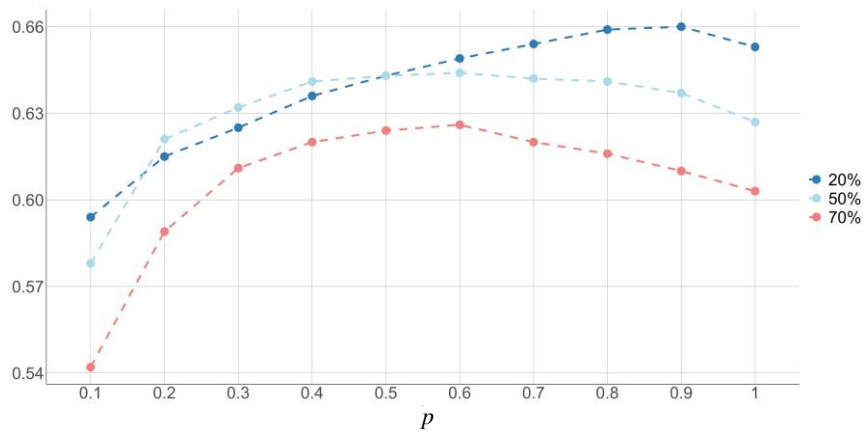

B

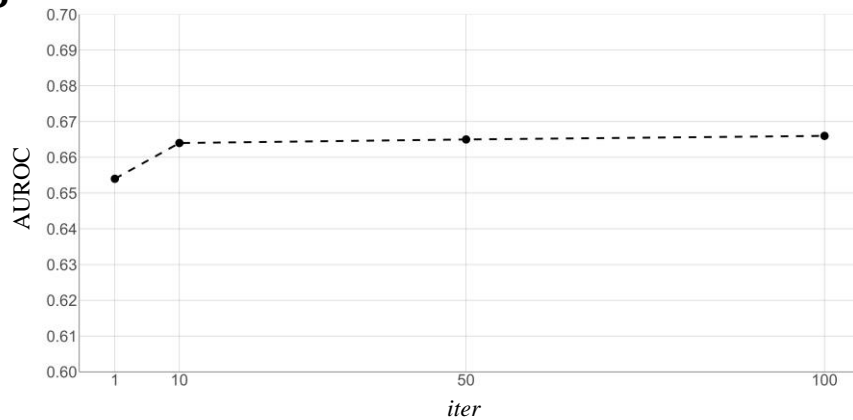

Supplement: Supplementary file 6 — Additional file 6: Supplementary Fig. 3. (A) The effect of the Bernoulli distribution parameter p on the performance of SRGS was examined. The Ecoli1 datasets with sample size of 100, two different gene sizes (50 and 100) and three different dropout rates (20, 50, and 70%) were used as representatives of simulated single-cell datasets with dropouts. The value of p was varied from 0.1 to 1 with a step size of 0.1, and the procedure was repeated for ten times. The mean of AUROCs among the ten times was shown. (B) The Ecoli1 dataset with sample size of 100, gene size of 50 and dropout rate of 50% was used as an example to check the effect of the number of iterations on the performance of SRGS. [file 12864_2022_9020_MOESM6_ESM.pdf]

mHSC-E

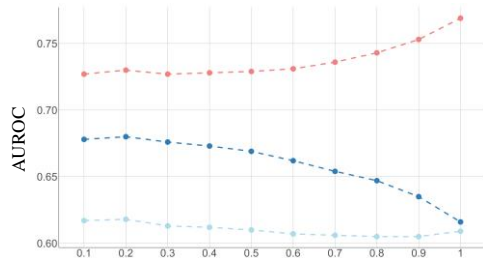

mHSC-GM

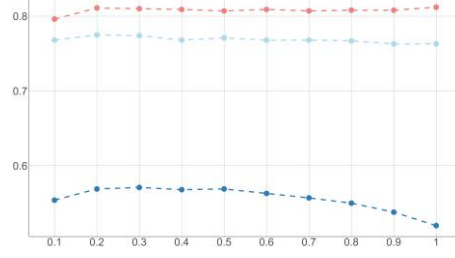

mHSC-L

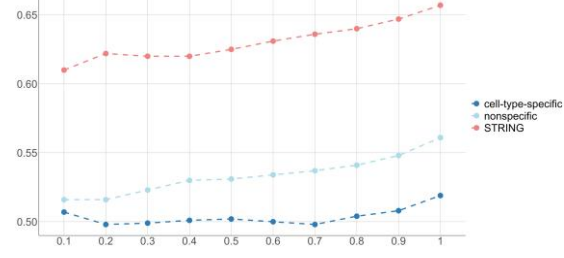

Top 500

hESC

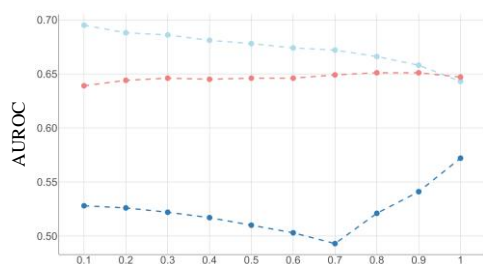

hHep

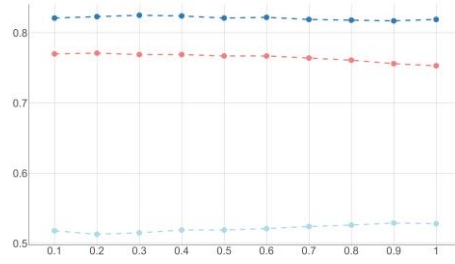

mDC

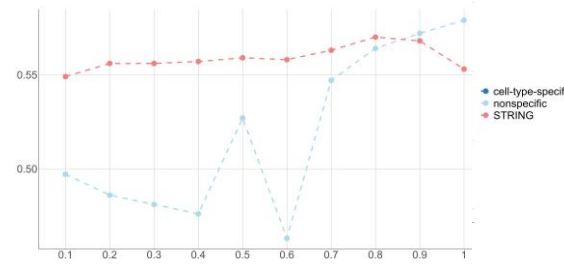

mHSC-E

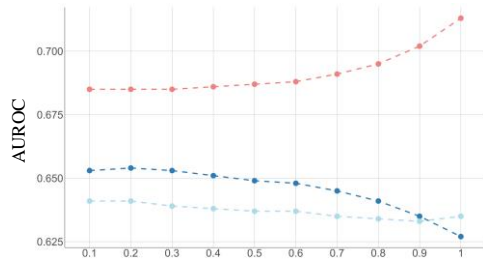

mHSC-GM

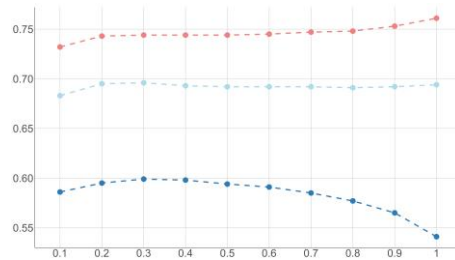

mHSC-L

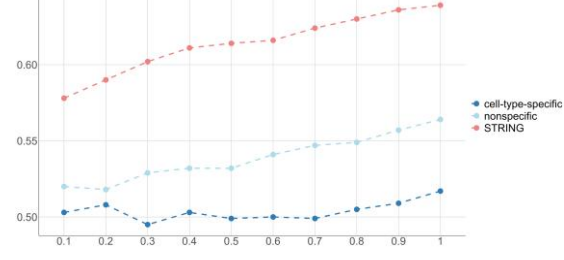

Top 1000

hESC

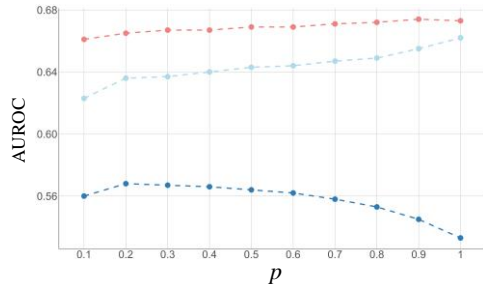

hHep

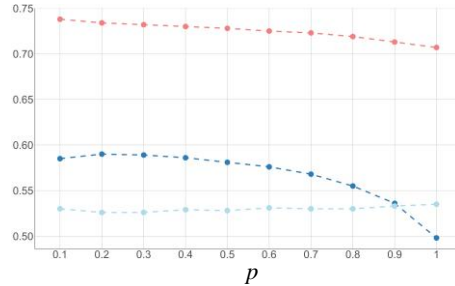

mDC

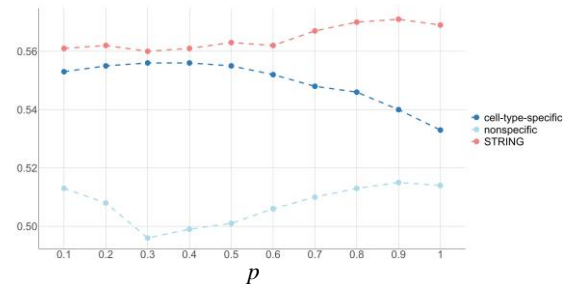

Supplement: Supplementary file 7 — Additional file 7: Supplementary Fig. 4. The selection of Bernoulli distribution parameter p for each experimental single-cell gene expression dataset (including mHSC-E, mHSC-GM, mHSC-L, mDC, hESC and hHEP) and each referenced ground-truth (including cell-type-specific ChIP-seq data, nonspecific ChIP-seq data, and STRING network) when top 500 and 1000 genes were used for GRN inference. [file 12864_2022_9020_MOESM7_ESM.pdf]

cell-type-specific

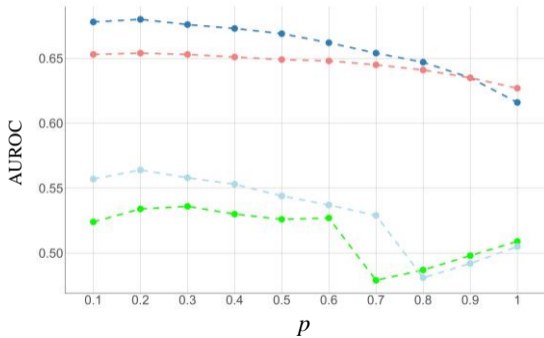

nonspecific

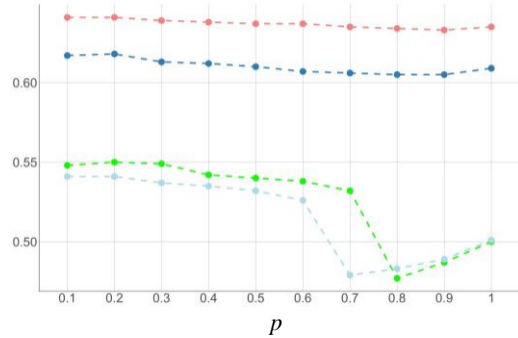

STRING

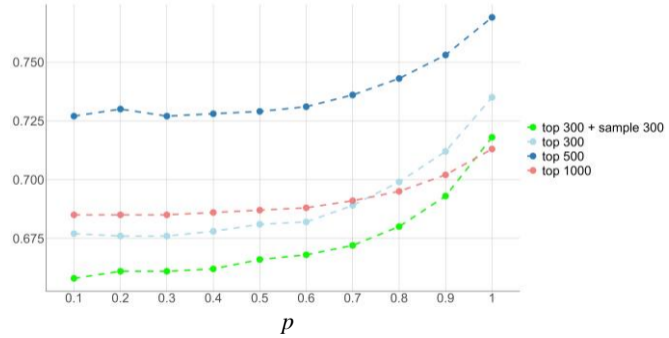

Supplement: Supplementary file 8 — Additional file 8: Supplementary Fig. 5. The selection of Bernoulli distribution parameter p when top 300 genes and also randomly selected 300 samples, top 300 genes, top 500 genes, and top 1000 genes were used. mHSC-E was used as a representative of experimental single-cell gene expression datasets to test. [file 12864_2022_9020_MOESM8_ESM.pdf]
